# Self-reported sexually transmitted infections among adolescent girls and young women in sub-Saharan Africa

**DOI:** 10.1093/inthealth/ihab088

**Published:** 2022-02-03

**Authors:** Louis Kobina Dadzie, Ebenezer Agbaglo, Joshua Okyere, Richard Gyan Aboagye, Francis Arthur-Holmes, Abdul-Aziz Seidu, Bright Opoku Ahinkorah

**Affiliations:** Department of Population and Health, University of Cape Coast, Cape Coast, Ghana; Department of English, University of Cape Coast, Cape Coast, Ghana; Department of Population and Health, University of Cape Coast, Cape Coast, Ghana; School of Public Health, University of Health and Allied Sciences, Ho, Ghana; Department of Sociology and Social Policy, Lingnan University, 8 Castle Peak Road, Tuen Mun, Hong Kong; Department of Estate Management, Takoradi Technical University, Takoradi, Ghana; Centre for Gender and Advocacy, Takoradi Technical University, Takoradi, Ghana; College of Public Health, Medical and Veterinary Sciences, James Cook University, Townsville, QLD, Australia; School of Public Health, Faculty of Health, University of Technology Sydney, Sydney, NSW, Australia

**Keywords:** adolescent girls, prevalence, self-reported sexually transmitted infections, sub-Saharan Africa, young women

## Abstract

**Background:**

Sexually transmitted infections (STIs) affect individuals of all ages, but adolescent girls and young women are disproportionately affected. We examined the prevalence and factors associated with self-reported STIs (SR-STIs) among adolescent girls and young women in sub-Saharan Africa (SSA).

**Methods:**

Demographic and Health Survey data of 27 sub-Saharan African countries were used for the study. The sample size comprised 68944 adolescent girls and young women (15–24 y of age). The outcome variable was SR-STIs. Multilevel binary logistic regression analysis was performed to identify factors associated with SR-STIs.

**Results:**

On average, the prevalence of SR-STIs among adolescent girls and young women in SSA was 6.92%. The likelihood of SR-STIs was higher among young women aged 20–24 y (adjusted odds ratio [aOR] 1.36 [confidence interval {CI} 1.27 to 1.46]), those not married (aOR 1.64 [CI 1.51 to 1.79]), those working (aOR 1.20 [CI 1.12 to 1.27]), those whose age at first sex was ≤19 y (aOR 1.99 [CI 1.80 to 2.20]), those with two or more sex partners (aOR 1.56 [CI 1.35 to 1.80]), those who listened to radio (aOR 1.26 [CI 1.17 to 1.35]), those in urban areas (aOR 1.42 [CI 1.30 to 1.51]) and those with a wealth index of rich (aOR 1.28 [CI 1.17 to 1.40]) compared with their counterparts. In contrast, those with a primary (aOR 0.86 [CI 0.78 to 0.94]) or secondary/higher level of education (aOR 0.83 [CI 0.75 to 0.92]) compared with those with no formal education and those who were exposed to television (aOR 0.90 [CI 0.84 to 0.98]) compared with those who were not exposed were less likely to report STIs.

**Conclusions:**

Our findings demonstrate the need for countries in SSA to commit towards reducing the incidence of STIs. Community-based health educational programs are required to intensify the awareness of STIs and their prevention in various sub-Saharan African countries considering the factors that expose adolescent girls and young women to STIs.

## Introduction

Over the past 2 decades, sexually transmitted infections (STIs), including chlamydia, gonorrhoea, syphilis and human immunodeficiency virus (HIV), have been increasing and have become a public health concern for both developed and developing countries.^1–5^ STIs caused by sexual contact, including vaginal, oral and anal sex, have a great impact on the sexual and reproductive health of men and women age 15–49 y.^6–10^ STIs are a major global cause of infertility, acute illness, long-term disability and mortality, with severe medical and psychological consequences.^1^ Studies also show that STIs result in other negative health outcomes, including HIV/acquired immunodeficiency syndrome (AIDS), ectopic pregnancy, eye defects and pelvic inflammatory diseases.^11,12^

Globally, more than 1 million people contract STIs daily.^13,14^ In 2018, the World Health Organization reported 376 million new infections of four STIs—chlamydia, gonorrhoea, syphilis and trichomoniasis—among people ages 15–49 y in 2016.^14^ Prevalence rates of STIs vary from region to region, country to country and between urban and rural populations. The infections are more prevalent in Asia and sub-Saharan Africa (SSA), but SSA contributes a greater proportion of the cases of STIs every year, which is estimated at approximately 93 million.^15^

STIs affect individuals of all ages,^16^ but adolescent girls and young women are disproportionately impacted due to their high-risk sexual behaviours, including unprotected sex.^17^ Studies have shown that adolescent girls and young women are at a higher risk of acquiring STIs because of social circumstances and increased risk-taking.^8,17,18^ Self-reported STIs (SR-STIs) allows health facilities to provide specific prevention and control interventions to tackle the spread of infections.^19,20^

Yet, for SSA, studies that have focused on SR-STIs have failed to examine the factors associated with STIs among adolescent girls and young women.^8,9,15,21^ For example, Torrone et al.^8^ used combined data from 18 prospective HIV prevention studies in SSA to investigate the association between hormonal contraceptive use and contraction of HIV. Other studies on STIs in SSA have examined the prevalence and the associated factors of SR-STIs among men,^15^ men who have sex with men and transgender women.^21,22^ For Seidu et al.,^15^ sexually active men ages 25–34 y were more likely to report STIs compared with those age ≥45 y.

Given this gap in research knowledge, we utilized recent data from sub-Saharan African countries’ Demographic and Health Surveys (DHS) to examine the prevalence and associated factors of SR-STI among adolescent girls and young women in SSA. Findings from this study will strengthen our understanding of STIs and factors associated with SR-STIs among adolescent girls and young women in SSA. This study will also provide information on the country-level variations in the prevalence and factors associated with SR-STIs among adolescent girls and young women in this region.

## Methods

### Data source

Data for this study were obtained from the recent DHS of 27 sub-Saharan African countries. These surveys, which had information on SR-STIs, were conducted from 2010 to 2018. Given the focus of the present study, we used data from women's file which also contains information on adolescent girls and young women (ages 15–24 y) from the various countries. Generally, as a nationally representative survey, the DHS is globally undertaken in over 85 low- and middle-income countries. With regard to its sampling method and focus, the DHS adopts a two-stage stratified sampling protocol, focusing on essential maternal and child health markers and men's health, including SR-STIs.^23^ The dataset is freely accessible at https://dhsprogram.com/data/available-datasets.cfm. Details of the DHS methodology have been reported elsewhere.^23,24^ The present study used a sample of 68944 adolescent girls and young women in SSA who had ever had sex in the past 12 months and had complete information on all the variables of interest (Table 1). We followed the Strengthening the Reporting of Observational Studies in Epidemiology statement in conducting this research.

**Table 1.** Description of study sample

Country	Weighted frequency	Weighted percentage
Benin 2017/2018	3719	5.39
Burkina Faso 2010	1709	2.48
Burundi 2016/2017	2030	2.94
Cameroon 2018	4280	6.21
Chad 2014/2015	3696	5.36
Comoros 2012	1669	2.42
Congo 2011/2012	1039	1.51
Cote D'Ivoire 2011/2012	683	0.99
DR Congo 2013/2014	3292	4.78
Ethiopia 2016	3045	4.42
Gabon 2012	2507	3.64
Gambia 2013	2376	3.45
Ghana 2014	2324	3.37
Guinea 2018	1600	2.32
Kenya 2014	1646	2.39
Lesotho 2014	516	0.75
Liberia 2013	2738	3.97
Malawi 2015/2016	6344	9.2
Mali 2018	2634	3.82
Mozambique 2011	2281	3.31
Namibia 2013	2255	3.27
Niger 2012	2054	2.98
Senegal 2010/2011	2559	3.71
Togo 2013/2014	1895	2.75
Uganda 2016	4840	7.02
Zambia 2018	3318	4.81
Zimbabwe 2015	1893	2.75
All countries	68 944	100

**Table 2.** Distribution of SR-STIs across the explanatory variables (weighted n=68 944)

			Had STI in the last 12 months, %		
Variables	Weighted frequency	Weighted percentage	No	Yes	p-Value
Age in 5-y groups					<0.001
15–19	24 462	35.5	93.8	6.2	
20–24	44 482	64.5	92.7	7.3	
Highest education level					<0.001
None	15 663	22.7	94.5	5.5	
Primary	22 582	32.8	93.8	6.2	
Secondary/higher	30 699	44.5	91.8	8.2	
Current marital status					<0.001
Not married	36 043	52.3	91.0	9.0	
Married	32 901	47.7	95.3	4.7	
Respondent's occupation					<0.001
Not working	31 547	45.8	93.6	6.4	
Working	37 397	54.2	92.6	7.4	
Age at first sex (years)					<0.001
≤19	54 695	79.3	92.2	7.8	
≥20	14 249	20.7	96.4	3.6	
Number of sex partners, excluding spouse, in last 12 months					<0.001
0	43 629	63.3	94.2	5.8	
1	23 101	33.5	91.6	8.4	
≥2	2214	3.2	85.3	14.7	
Condom use during last sex with most recent partner					0.018
No	56 854	82.5	92.9	7.1	
Yes	12 090	17.5	93.9	6.1	
Comprehensive HIV knowledge					<0.001
No	42 530	61.7	93.5	6.5	
Yes	26 414	38.3	92.5	7.5	
Ever been tested for HIV					0.68
No	28 322	41.1	93.1	6.9	
Yes	40 622	58.9	93.1	6.9	
Read newspaper or magazine					<0.001
No	54 025	78.4	93.5	6.5	
Yes	14 919	21.6	91.7	8.3	
Listen to radio					<0.001
No	28 443	41.3	94.0	6.0	
Yes	40 501	58.7	92.5	7.5	
Watch television					<0.001
No	38 114	55.3	94.0	6.0	
Yes	30 830	44.7	92.0	8.0	
Covered by health insurance					<0.001
No	64 933	94.2	93.2	6.8	
Yes	4011	5.8	91.2	8.8	
Place of residence					<0.001
Urban	27 833	40.4	90.7	9.3	
Rural	41 111	59.6	94.7	5.3	
Wealth index					<0.001
Poor	25 391	36.8	94.7	5.3	
Middle	13 620	19.8	94.0	6.0	
Rich	29 933	43.4	91.3	8.7	
Geographical subregion					<0.001
Southern Africa	2771	4.0	98.0	2.0	
Western Africa	24 291	35.2	93.4	6.6	
Central Africa	14 814	21.5	90.7	9.3	
Eastern Africa	27 068	39.3	93.6	6.4	

### Study variables

#### Outcome variable

SR-STIs among adolescent girls and young women was the outcome variable in this research. Adolescent girls and young women were asked whether they had a disease they acquired through sexual contact in the past 12 months (yes/no). This implies that the variable has a dichotomous outcome.

#### Explanatory variables

Sixteen explanatory variables made up of 13 individual-level variables and 3 contextual-level variables were included in this analysis. The individual-level variables were age (15–19 y, 20–24 y), education level (no education, primary, secondary/higher), marital status (married, not married), occupation (not working, working), age at first sex (≤19, ≥20), number of sexual partners in the last 12 months excluding the spouse (0, 1, ≥2), condom use during last sex with most recent partner (yes, no), comprehensive HIV and AIDS knowledge (yes, no), HIV testing (yes, no), exposure to mass media (newspaper, radio, TV) (yes, no) and health insurance coverage (yes, no). The contextual-level variables were place of residence (rural, urban), wealth index (poor, middle, rich) and subregion (western Africa, eastern Africa, central Africa, southern Africa). These factors were chosen based on their theoretical and empirical relationship with SR-STIs in previous studies.^23,24^

### Statistical analyses

Data analyses, which involved both descriptive and inferential analyses, were carried out using Stata version 14.0 (StataCorp, College Station, TX, USA). The descriptive statistics enabled us to characterize the adolescent girls and young women in the data. The data were weighted to account for sampling probability and non-response. Also, the data were adjusted using the SVY command in Stata to account for the complex survey design and robust standard errors. Pearson chi-sqaure analysis was conducted to select potential variables for the follow-up multilevel logistic regression analysis. At the bivariate analysis stage, due to multiple comparisons, we introduced the Bonferroni correction method.^25^ This was done by dividing the α rate (p=0.05) by the number of analyses performed (16 explanatory variables), that is, 0.05/16=0.003. Therefore, in the bivariate analysis, statistical significance was declared at p≤0.003. Variables with a p-value ≤0.003 in the bivariate analysis were included in the multilevel logistic regression model. Before fitting the final model, multicollinearity between the explanatory variables was checked (mean variance inflation factor [VIF] 1.35, minimum VIF 1.05, maximum VIF 2.01) and it was found to be satisfactory. The multilevel logistic regression analysis was performed to identify factors associated with SR-STIs. Four models were presented. Model 0 showed the variance in SR-STIs attributed to the clustering of the primary sampling units (PSUs) without the explanatory variables. Models 1 and 2 contained the individual- and contextual-level factors, respectively. The final model (model 3) had all the individual- and contextual-level factors. The Stata command melogit was used when fitting these models. The Akaike information criterion (AIC) test was used for model comparison. The descriptive results are presented as proportions while the regression results are presented as adjusted odds ratios (aORs) with 95% confidence intervals (CIs) and p-values.

### Ethical consideration

Since this study used secondary data that is publicly available, the authors did not need further ethics approval. However, permission to use the dataset was sought from MEASURE DHS. Also, appropriate authorizations and procedures applicable in the respective countries were followed and these are reported in the various national reports. Further information on ethics for the DHS is available at https://dhsprogram.com/Methodology/Protecting-the-Privacy-of-DHS-Survey-Respondents.cfm.

## Results

### Prevalence of SR-STIs among adolescent girls and young women in SSA

**Figure 1.**
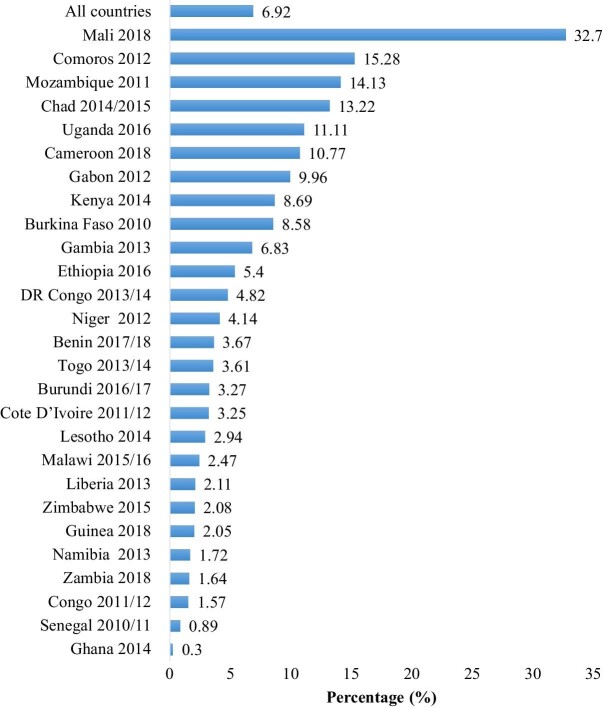
Prevalence of SR-STIs by country.

Figure 1 shows the prevalence of SR-STIs among adolescent girls and young women across 27 countries in SSA. On average, the prevalence of SR-STIs among adolescent girls and young women in SSA was 6.92%. In terms of cross-country variations, the prevalence ranged from 32.7% in Mali to 0.3% in Ghana.

### Sociodemographic characteristics and SR-STIs among adolescent girls and young women in SSA

At p≤0.003, all the explanatory variables showed statistically significant associations with SR-STIs among adolescent girls and young women in SSA, except condom use during last sex with the most recent partner and ever been tested for HIV. Specifically, a higher prevalence of STIs was recorded among those ages 20–24 y (7.3%); those with secondary or higher education (8.2%); those not married (9.0%); those who were working (7.4%); those ≤19 y of age at first sex (7.8%); those with two or more partners (14.7%); those with comprehensive knowledge of HIV (7.5%); those who read newspapers (8.3%), listened to radio (7.5%) or watched television (8.0%); those covered by health insurance (8.8%); those residing in urban areas (9.3%); those with a wealth index of rich (8.7%) and adolescent girls and young women in central Africa (9.3%) compared with their counterparts in the other variable categories.

### Multilevel logistic regression results on the factors associated with SR-STIs among adolescent girls and young women in SSA

Table 3 shows the results from the multilevel logistic regression analysis of the factors associated with SR-STIs among adolescent girls and young women in SSA. The final model, which contains all the explanatory variables, revealed that in terms of individual-level factors, the likelihood of SR-STIs was higher among adolescent girls and young women ages 20–24 y (aOR 1.36 [CI 1.27 to 1.46]) compared with those ages 15–19 y. Again, those not married (aOR 1.64 [95% CI 1.51 to 1.79]), those working (aOR 1.20 [95% CI 1.12 to 1.27]) and those whose age at first sex was ≤19 y (aOR 1.99 [95% CI 1.80 to 2.20]) were more likely to report an STI as compared with those who were married, those not working and those with an age at first sex of ≥20 y, respectively. Also, those with two or more sex partners (aOR 1.56 [95% CI 1.35 to 1.80]) compared with those with no sex partner and those who listened to radio (aOR 1.26 [95% CI 1.17 to 1.35]) compared with those who did not listen to radio were more likely to self-report STI. On the other hand, those with a primary (aOR 0.86 [95% CI 0.78 to 0.94) or secondary/higher level of education (aOR 0.83 [95% CI 0.75 to 0.92]) compared with those with no formal education and those who watched television (aOR 0.90 [95% CI 0.84 to 0.98]) compared with those who did not watch television were less likely to report an STI. For the contextual factors, those who lived in urban areas (aOR 1.42 [95% CI 1.30 to 1.51]) compared with rural residents, those a wealth index of rich (aOR 1.28 [95% CI 1.17 to 1.40]) compared with those with a wealth index of poor and those in all subregions compared with those in southern Africa were more likely to report an STI.

**Table 3.** Predictors of SR-STIs among adolescent girls and young women in SSA

Variables	Model 0	Model 1, aOR (95% CI)	Model 2, aOR (95% CI)	Model 3, aOR (95% CI)
Age in 5-y groups				
15–19		1 (1.00 to 1.00)		1 (1.00 to 1.00)
20–24		1.40*** (1.31 to 1.50)		1.36*** (1.27 to 1.46)
Highest education level				
None		1 (1.00 to 1.00)		1 (1.00 to 1.00)
Primary		0.87** (0.79 to 0.96)		0.86** (0.78 to 0.94)
Secondary/higher		0.98 (0.89 to 1.07)		0.83*** (0.75 to 0.92)
Current marital status				
Not married		1.75*** (1.60 to 1.90)		1.64*** (1.51 to 1.79)
Married		1 (1.00 to 1.00)		1 (1.00 to 1.00)
Respondents occupation				
Not working		1 (1.00 to 1.00)		1 (1.00 to 1.00)
Working		1.19*** (1.11 to 1.26)		1.20*** (1.12 to 1.27)
Age at first sex (years)				
≤19		2.01*** (1.82 to 2.22)		1.99*** (1.80 to 2.20)
≥20		1 (1.00 to 1.00)		1 (1.00 to 1.00)
Number of sex partners, excluding spouse, in the last 12 months				
0		1 (1.00 to 1.00)		1 (1.00 to 1.00)
1		0.97 (0.89 to 1.05)		0.91* (0.84 to 0.99)
≥2		1.71*** (1.48 to 1.96)		1.56*** (1.35 to 1.80)
Comprehensive HIV knowledge				
No		1 (1.00 to 1.00)		1 (1.00 to 1.00)
Yes		1.07* (1.00 to 1.14)		1.06 (0.99 to 1.13)
Read newspaper or magazine				
No		1 (1.00 to 1.00)		1 (1.00 to 1.00)
Yes		0.97 (0.90 to 1.05)		0.99 (0.92 to 1.07)
Listen to radio				
No		1 (1.00 to 1.00)		1 (1.00 to 1.00)
Yes		1.18*** (1.10 to 1.26)		1.26*** (1.17 to 1.35)
Watch television				
No		1 (1.00 to 1.00)		1 (1.00 to 1.00)
Yes		1.12*** (1.05 to 1.20)		0.90** (0.84 to 0.98)
Covered by health insurance				
No		1 (1.00 to 1.00)		1 (1.00 to 1.00)
Yes		1.05 (0.94 to 1.19)		1.10 (0.97 to 1.23)
Residence				
Urban			1.54*** (1.42 to 1.66)	1.42*** (1.30 to 1.54)
Rural			1 (1.00 to 1.00)	1 (1.00 to 1.00)
Wealth index				
Poor			1 (1.00 to 1.00)	1 (1.00 to 1.00)
Middle			1.02 (0.92 to 1.11)	1.00 (0.91 to 1.10)
Rich			1.27*** (1.17 to 1.39)	1.28*** (1.17 to 1.40)
Geographical subregion				
Southern Africa			1 (1.00 to 1.00)	1 (1.00 to 1.00)
Western Africa			3.07*** (2.32 to 4.05)	2.37*** (1.79 to 3.14)
Central Africa			4.74*** (3.59 to 6.26)	3.48*** (2.62 to 4.61)
Eastern Africa			3.51*** (2.66 to 4.62)	2.41*** (1.82 to 3.20)
Random effects model				
PSU variance (95% CI)	0.27 (0.23 to 0.33)	0.26 (0.21 to 0.31)	0.27 (0.22 to 0.32)	0.26(0.21 to 0.31)
ICC	0.0765	0.0727	0.0751	0.0723
Wald χ^2^	Ref	861.60***	581.52***	1168.69***
Model fitness				
log-likelihood	−17 047.5	−16 948.16	−16 731.03	−16 402.99
AIC	34 098.98	33 197.06	33 478.06	32 847.97
BIC	34 117.25	33 334.07	33 551.13	33 039.78
Number of clusters	1479	1479	1479	1479

Exponentiated coefficients.

*p<0.05, **p<0.01, ***p<0.001.

Ref: reference category; PSU: primary sampling unit; ICC: intraclass correlation; BIC: Bayesian information criterion.

## Discussion

This study examined the prevalence and factors associated with SR-STIs among adolescent girls and young women in SSA. We found that overall there was a relatively low prevalence of SR-STIs (6.92%) among adolescent girls and young women from the 27 sub-Saharan African countries included in the study. Nevertheless, this prevalence is higher than that found among men (3.8%) in a related study by Seidu et al.^15^ Women are known to have better health-seeking behaviours compared with men, which may explain the high prevalence of SR-STIs among adolescent girls and young women in this study.^26^ The SR-STI prevalence in this study implies that there is much to be done in SSA to attain the global health strategy for STIs, which envisions a world where everybody has free and easy access to STI prevention and treatment services.^27^

Even though the overall prevalence across the countries in this study was low, we found intercountry variations in the prevalence of SR-STIs, with adolescent girls and young women from Mali reporting the highest prevalence while those from Ghana reported the lowest prevalence of SR-STIs. The results are substantiated by recent findings by Smolak,^28^ who also reported a higher prevalence of STIs and HIV among Malian women. This could possibly be explained by the pervasiveness of some sociocultural practices, such as female genital mutilation in Mali, which has been found to be linked with an increased likelihood of engaging in multiple sexual partnerships, a factor that increases the risk of contracting STIs.^29^ As reported in Ghana, good access to sexual and reproductive health services could account for the low prevalence of SR-STIs among Ghanaian women.^30^ Other possible reasons for the wide difference in the prevalence of SR-STIs in Ghana and Mali could be country-level variations in the distribution of factors associated with SR-STIs, such as age and socio-economic status, which were found to have significant associations with SR-STIs in our study.

We recorded a statistically significant association between age and SR-STIs among adolescent girls and young women in SSA. Specifically, women ages 20–24 years were more likely to report STIs compared with girls of younger ages. This is in agreement with previous studies that found women ages 20–24 y have a higher risk of STIs.^31^ A plausible explanation for this result may be found in the study by Sathiyasusuman,^31^ which revealed that women ages 20–24 y are more likely to engage in multiple sexual partnerships, predisposing them to a higher likelihood of contracting an STI. Multiple sexual partnerships, whether serial or concurrent, have been found to be significantly associated with the risk of STIs among women.^32^ It is therefore not surprising that women with two or more sexual partners had a higher risk of reporting STIs.

Available evidence suggests that being married is a protective factor against STIs among adolescent girls and young women.^33–35^ Our findings confirm this. We found that women who are married are less likely to report STIs compared with those who are not married. Perhaps this could be attributed to the premium that Africans place on marriage. A married person is supposed to live up to societal and cultural expectations,^36^ and in the case of women, they are expected to be humble and not engage in acts of infidelity. Therefore it is not expected that married adolescent girls and young women would engage in multiple sexual partnerships. Another plausible explanation for the lower likelihood of SR-STIs among married adolescent girls and young women could be that in many countries in SSA, like Nigeria^37^ and Ghana,^38^ the phenomenon of compulsory premarital STI testing, including HIV/AIDS testing, has become common. Thus married adolescent girls and young women know their status before marriage. Therefore it is expected that the odds of SR-STIs would be low among married adolescent girls and young women. However, our findings are not in agreement with previous findings that married individuals have higher likelihoods of reporting STIs since they are more likely to report non-use of condoms.^39^

Our findings also suggest that adolescent girls and young women who reside in rural areas are less likely to report STIs. Living in an urban area comes with a host of concomitant risks. The anonymity of being a migrant increases one's likelihood of engaging in risky sexual behaviours, including commercial sex work and multiple sexual partners.^40^ For instance, reports from Ghana indicate that in rural areas, women are less exposed to risky sexual behaviours that contribute to STIs; however, when they migrate to urban areas, they work as head porters, known as *kayayei* in Ghana, sleeping in the open and becoming targets of rapists who have unprotected sex with them.^41^ Also, adolescent girls and young women living in rural areas are marginalized and disempowered and face geographical barriers in terms of accessing STI knowledge and services, including testing,^42,43^ thus explaining the low odds of SR-STIs among rural adolescent girls and young women in this study.

Access to the media (i.e. radio) was found to be significantly associated with the likelihood of reporting STIs among adolescent girls and young women in SSA. This study provided evidence showing that having greater access to the media increased the odds of reporting STIs. The result is in line with evidence from Nigeria^44^ showing that the odds of reporting an STI is positively associated with the frequency of access to the media. This could be explained from the perspective that often these media platforms show health promotional messages that encourage people to go for testing and by so doing many adolescent girls and young women become aware of their STI status.^45^ Another reason for this finding could be that some newspapers carry sexually explicit content that could entice adolescent girls and young women to engage in risky sexual behaviours, which increases their chances of reporting STIs.^15,44^ This supports the finding in the present study that adolescent girls and young women in rich households had a higher risk of reporting STIs. A wealth index of rich implies that they can afford access to mass media that has become a conduit through which STI screening and testing are promoted.

## Strengths and limitations

We used data from the recent DHS, which employed robust methodologies in its data collection and has been validated by several studies. Also, the use of nationally representative data ensures that our findings are generalizable to adolescent girls and young women in SSA. Nonetheless, the study had some limitations. The use of secondary data limited our analysis to variables that were present in the datasets. Therefore important variables such as the effect of culture and patriarchal norms that may be associated with the risk of reporting STIs among women could not be assessed. Also, due to the cross-sectional design employed by the DHS, causal inferences cannot be made from the findings. Moreover, the outcome variable was based on self-reports, which are prone to recall bias. Therefore readers are cautioned to interpret the findings taking these limitations into account. Finally, the large sample size may affect conclusions on associations since any small association could be found to be statistically significant. However, this was addressed to some extent by using a multilevel analysis.

## Conclusions

Our study found a relatively low prevalence of SR-STIs among adolescent girls and young women in SSA. Despite the low prevalence, much effort will be needed to increase the ability of SSA countries to attain the global health strategy for STIs prevention and treatment. Our findings demonstrate the urgent need for countries within SSA to commit to further reductions in the prevalence of STIs. In particular, countries like Mali, which recorded a high prevalence of STIs, should make conscious efforts to target priority populations that are at higher risk of STIs (i.e. younger women, those in the urban areas, those with a wealth index of rich and those engaged in multiple sexual partnerships). From our findings, we conclude that the media plays a critical role in promoting SR-STIs. Therefore STI education through the media should be strengthened in order for adolescent girls and young women to seek STI testing. Public education on the effects of multiple sexual partnership should also be increased across the region. Future studies should explore the effects of age and socio-economic status on the awareness of STI symptoms.

## Data Availability

https://dhsprogram.com/data/available-datasets.cfm.
